# Spatiotemporal Structure of Molecular Evolution of H5N1 Highly Pathogenic Avian Influenza Viruses in Vietnam

**DOI:** 10.1371/journal.pone.0008631

**Published:** 2010-01-08

**Authors:** Margaret A. Carrel, Michael Emch, R. Todd Jobe, Aaron Moody, Xiu-Feng Wan

**Affiliations:** 1 Department of Geography, University of North Carolina-Chapel Hill, Chapel Hill, North Carolina, United States of America; 2 Department of Basic Sciences, College of Veterinary Medicine, Mississippi State University, Mississippi State, Mississippi, United States of America; University of Bristol, United Kingdom

## Abstract

**Background:**

Vietnam is one of the countries most affected by outbreaks of H5N1 highly pathogenic avian influenza viruses. First identified in Vietnam in poultry in 2001 and in humans in 2004, the virus has since caused 111 cases and 56 deaths in humans. In 2003/2004 H5N1 outbreaks, nearly the entire poultry population of Vietnam was culled. Our earlier study (Wan *et al.*, 2008, *PLoS ONE*, **3**(10): e3462) demonstrated that there have been at least six independent H5N1 introductions into Vietnam and there were nine newly emerged reassortants from 2001 to 2007 in Vietnam. H5N1 viruses in Vietnam cluster distinctly around Hanoi and Ho Chi Minh City. However, the nature of the relationship between genetic divergence and geographic patterns is still unclear.

**Methodology/Principal Findings:**

In this study, we hypothesized that genetic distances between H5N1 viruses in Vietnam are correlated with geographic distances, as the result of distinct population and environment patterns along Vietnam's long north to south longitudinal extent. Based on this hypothesis, we combined spatial statistical methods with genetic analytic techniques and explicitly used geographic space to explore genetic evolution of H5N1 highly pathogenic avian influenza viruses at the sub-national scale in Vietnam. Our dataset consisted of 125 influenza viruses (with whole genome sets) isolated in Vietnam from 2003 to 2007. Our results document the significant effect of space and time on genetic evolution and the rise of two regional centers of genetic mixing by 2007. These findings give insight into processes underlying viral evolution and suggest that genetic differentiation is associated with the distance between concentrations of human and poultry populations around Hanoi and Ho Chi Minh City.

**Conclusions/Significance:**

The results show that genetic evolution of H5N1 viruses in Vietnamese domestic poultry is highly correlated with the location and spread of those viruses in geographic space. This correlation varies by scale, time, and gene, though a classic isolation by distance pattern is observed. This study is the first to characterize the geographic structure of influenza viral evolution at the sub-national scale in Vietnam and can shed light on how H5N1 HPAIVs evolve in certain geographic settings.

## Introduction

Highly pathogenic H5N1 avian influenza viruses (HPAIVs), first isolated in Guangdong, China in 1996 [1], have spread from southern China across Southeast, East and Central Asia, to the Middle East, Europe and Africa [2]–[5]. Millions of domestic and wild birds have either died or been culled because of the outbreaks caused by these H5N1 viruses. H5N1 HPAIVs have also proven to be highly lethal in humans: 262 fatalities from 440 reported cases, representing a mortality rate of about 60%, as of August 31, 2009 [6]. Recently emerging family clusters in Indonesia and Pakistan have increased concern about human-to-human transmission of H5N1 viruses [7], [8], an essential step for epidemics and pandemics in human populations, which has not yet occurred. These human cases of H5N1 infection have raised a great concern for possible emergence of another novel influenza virus from reassortments between H5N1 HPAIVs and the 2009 H1N1 pandemic virus, which probably emerged in Mexico and spread rapidly through human-to-human transmissions and is causing the ongoing pandemic [9]. Such a reassortment between H5N1 and H1N1 viruses could increase the mortality and severity of the current influenza pandemic.

While H5N1 HPAIVs are geographically widespread, some countries have seen greater incidence in poultry and humans than others. H5N1 influenza in Vietnam was first identified in poultry in 2001 and in humans in 2004 [10], and has since caused 111 cases and 56 deaths in humans, as of August 31, 2009. During the 2003/2004 H5N1 outbreaks, nearly the entire poultry population of Vietnam was culled.

Phylogeographic studies reveal the potential evolutionary and migration history of H5N1 viruses. Reassortment events among gene segments derived from A/turkey/England/50–92/1991 (H5N1)-like HA gene and low pathogenic avian influenza viruses probably occurred in southern China in the early 1990s [11]–[14]. Since that time, H5 HA genes underwent dramatic changes: at least 10 distinct major clades and even more sub-clades have emerged during the past decade [15]. Frequent reassortments are occurring not only between H5N1 and other subtypes of AIVs but also within H5N1 AIVs [10], [12], [14]. Analyses of the evolutionary history of H5N1 viruses in Vietnam show close links with viruses in southern China [16] and suggest that introductions took place along the shared border between Vietnam and Yunnan and Guangxi Provinces [14], [17].

Since 2001, at least six clades/subclades of H5 HPAIV HA (Clade 0, 1, 2.3.2, 2.3.4, 3, and 5) and nine reassortants of H5N1 HPAIV emerged in Vietnam [10], [18], [19]. These H5N1 viruses formed two phylogenetic clusters across both northern and southern Vietnam after they were introduced into northern Vietnam and spread to southern Vietnam [10], [16]. Prior analysis of the geographic spread of evolving influenza viruses across Vietnam has been more descriptive than statistical. In this study, we sought to determine how location and spread of H5N1 HPAIVs through geographic space interacted with viral evolution in Vietnam between 2003 and 2007, using spatial statistics to map potential correlative changes between geographic and genetic distances.

The geography of Vietnam, as well as the genetic history of H5N1 avian influenza in the country, makes it particularly appropriate for a spatial analysis of H5N1 evolution. Vietnam is long (north-south) and narrow (east-west). Human population densities are highest around Hanoi and the Red River delta in the north and Ho Chi Minh City and the Mekong River delta in the south. Poultry densities are also higher in these areas. There also exist distinct regional differences in economic and agricultural patterns between the north and south. These patterns allow us to easily characterize regional variation between the north and the south. H5N1 viruses isolated in Vietnam that belong to Clade 1 genotype VN3 (Genotype Z) are from a single introduction [16], [17], show extreme phylogenetic clustering [14], [20], and have all eight genes descendant from one progenitor virus, A/duck/Hong Kong/821/2002 (H5N1) (HK821-like virus) [10]. The possibility of genes isolated at the same geographic point but derived from different precursor viruses, leading to spurious conclusions about interactions between geographic and genetic distance, are thus diminished.

Our results show that genetic evolution of VN3 (HK821-like) H5N1 viruses in Vietnamese domestic poultry is highly correlated with the location of those viruses in geographic space. This correlation varies by scale, time and gene, though a classic isolation by distance pattern is observed. This study is the first to characterize the geographic structure of influenza viral evolution at the sub-national scale in Vietnam, and can shed light on how H5N1 HPAIVs evolve in certain geographic settings. It also lends strength to the supposition that domestic bird populations are primary drivers of influenza viral evolution, although more studies need to be carried out to assess the potential roles of wild birds and domestic animals (e.g. pigs) in facilitating influenza genetic diversity.

## Results

### Bi-Modal Influenza Genetic-Geographic Distance Distribution

**Figure 1:**
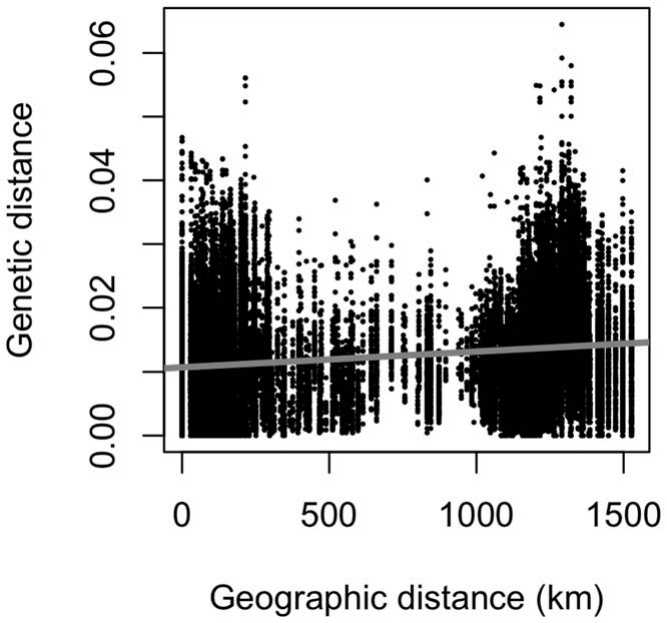
Genetic versus geographic distance of HK821-like HPAIVs in Vietnam. The least squares line is plotted in grey.

Visual data diagnostics revealed that the frequency distribution of geographic distance between case pairs was bi-modal with a cluster of relatively short and another of relatively long distances (Figure 1). This bi-modal pattern is linked to the distribution of H5N1 incidences in northern or southern Vietnam, with few isolates obtained in the central regions of the country. The distribution of genetic distances is also somewhat bi-modal, with case-pairs at both ends of the geographic scale exhibiting small and large genetic distances.

**Figure 2:**
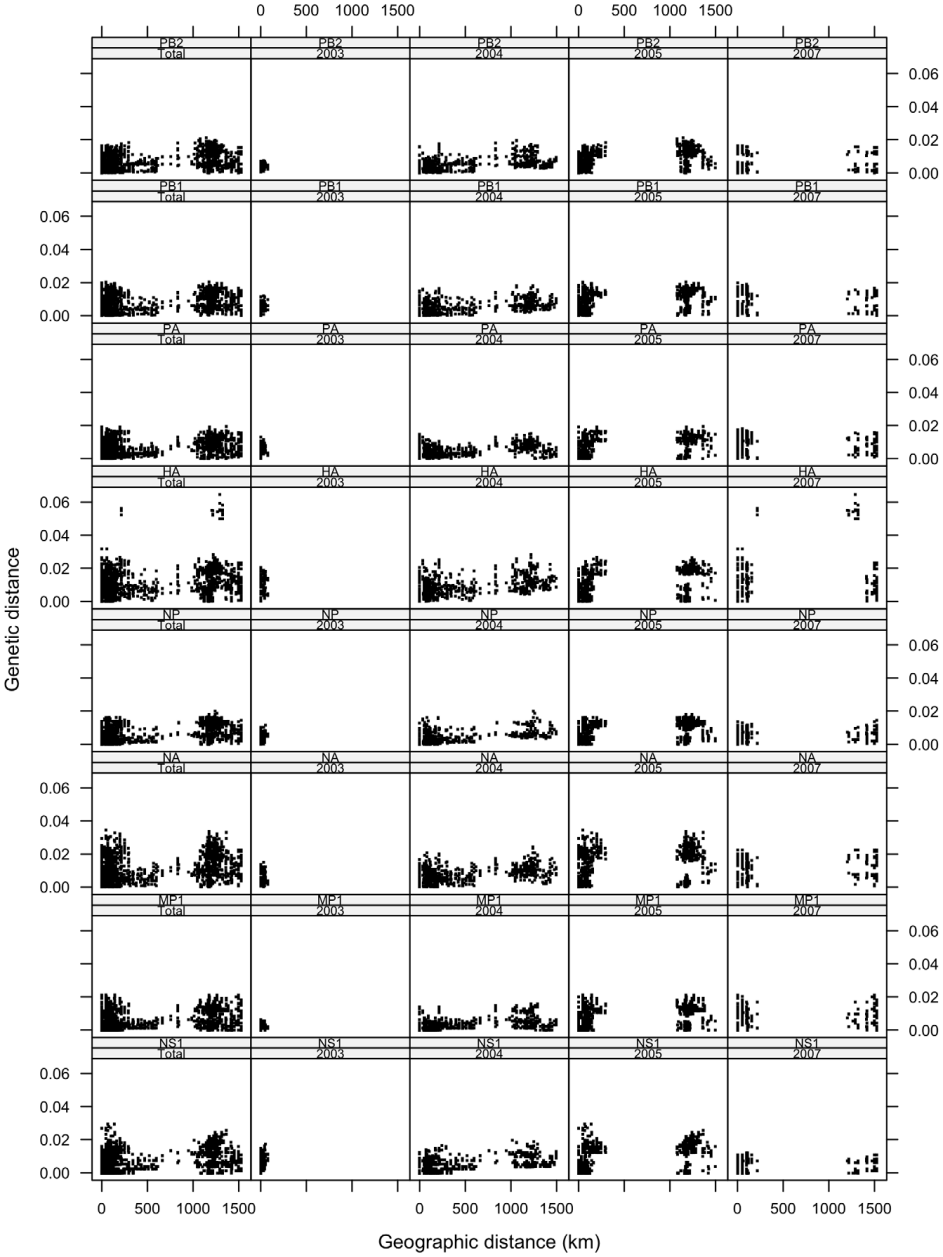
Genetic versus geographic distance of HK821-like HPAIVs, stratified by year and gene segment.

Individual gene scatterplots stratified by year revealed a marked difference in patterns by year and gene (Figure 2). Specifically, the bi-modal pattern of distances emerges for only specific year and gene combinations. In 2003 across all eight genes, the case pairs are all clustered at the low end of the geographic scale. This reflects a characteristic of our dataset, in which all 2003 cases occurred in northern Vietnam, geographically close to one another. In 2004 there is greater spread of points across the geographic scale, but relatively little across the genetic scale. By 2005, the bi-modal nature of influenza occurrence in the northern and southern regions of Vietnam is established and case pair distances cluster at the high and low ends of the geographic scale. Genetic distance between case pairs is higher in 2005 and 2007 than in the first two years of the dataset, an indication that these influenza viruses have evolved as local epidemic strains in domestic poultry after sweeping over Vietnam during the 2003–2004 outbreaks.

### Within- Versus Between-Region Genetic Variation

**Figure 3:**
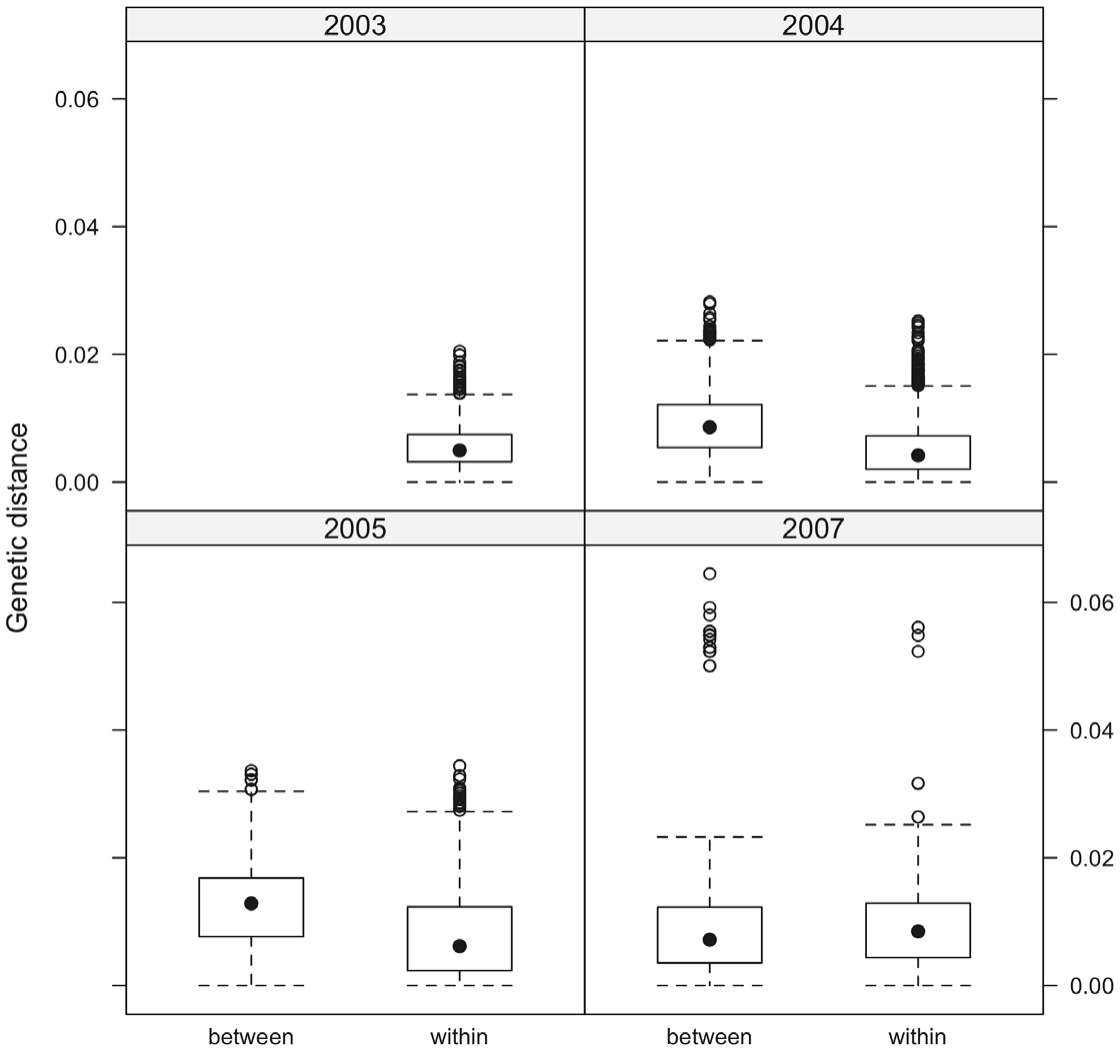
Boxplots of genetic versus geographic distance for the within- and between-region pairs of H5N1 HPAIVs in Vietnam. The solid black circle is the median genetic distance in each grouping. Hollow circles represent outliers.

Three-way ANOVA regressions (Table 1) indicate strongly significant main effects, two-way and three-way interactions within the dataset. The sampling unit for this ANOVA was pairs of observations. The response was genetic distance, and there were three binary predictors: gene, year, and region. These predictors were coded true when the observations pair had the same value, and coded false when the pair had different values. Though the strict ANOVA assumption of independence of observations is not met because distance matrices are based upon paired observations, this analysis provides an initial look at the trends present in the data and from which we expanded into more appropriate Mantel regressions that account explicitly for autocorrelation. The two-way interaction between region and year, controlling for gene, indicates that relative genetic distance between regions changes as you look across years (Figure 3). In 2003, when all cases were detected solely in northern Vietnam, there are no between-region case pairs, and the genetic distance in within-region case pairs is quite low. In 2004 and 2005, genetic distances among between-region cases are higher than within-region cases. By 2007, however, genetic distances are similar for the within- and between-region categories. The significant three-way interaction (Table 1) indicates that genetic variation in Vietnamese HK821-like cases is systematically different between regions, years and gene segments, and that these three factors interact in ways that affect genetic distances between virus case pairs. While the true significance of the relationships tested with the three-way ANOVA is masked by the non-independence of observations, the ANOVA results and the relationships plotted in Figure 3 led us to believe that interesting correlations existed between genetic and geographic distances among H5N1 viruses in Vietnam. Mantel test results, reported below, overcome the non-independence of matrix observations, and allowed us to accurately assess the relationship between geography and molecular evolution.

**Table 1:** Three-way ANOVA of genetic distance between case pairs of H5N1 highly pathogenic avian influenza viruses by region, segment (Gene), and year.

*Source*	*df*	*SumSquare*	*MeanSquare*	*F*	*p-value*
Region	1	0.07	0.07	2099.99	0.0000
Gene	7	0.06	0.01	293.44	0.0000
Year	1	0.01	0.01	471.87	0.0000
Region:Gene	7	0.00	0.00	14.23	0.0000
Region:Year	1	0.00	0.00	115.13	0.0000
Gene:Year	7	0.01	0.00	30.77	0.0000
Region:Gene:Year	7	0.00	0.00	11.44	0.0000
Residuals	16840	0.52	0.00		

### Significant Correlation between Genetic and Geographic Distances of Gene Segments

The results of the Mantel tests for correlation among matrices indicated significant, positive correlations between geographic and genetic distance for all eight influenza gene segments (Table 2). The PB2 and NS genes had the highest correlations.

**Table 2:** Mantel tests measuring correlation of geographic and genetic distances for H5N1 highly pathogenic avian influenza viruses from 2003 to 2007.

*Gene*	*Mantel r*	*p-value*	*CI 2.5%*	*CI 97.5%*
PB2	0.2310	0.001	0.2011	0.2678
PB1	0.1758	0.001	0.1509	0.2062
PA	0.2101	0.001	0.1800	0.2418
HA	0.1957	0.001	0.1698	0.2284
NP	0.2082	0.001	0.1779	0.2416
NA	0.2167	0.001	0.1865	0.2525
MP1	0.1951	0.001	0.1658	0.2300
NS1	0.2864	0.001	0.2565	0.3279

Correspondingly to the ANOVA results reported above, the multiple regressions using Mantel tests (MRM) indicated significant effects of geographic distance between viruses on genetic distance, while controlling for the effect of temporal distances, for all eight genes (Table 3). Simultaneously, year effects were shown for all genes while controlling for the effect of geographic distance. The R^2^ in the MRM analysis are also all statistically significant, though much higher for some gene segments (HA) than others (NA).

**Table 3:** MRM results of genetic distance on spatial lag and temporal lag for the VN3 subset.

*Gene*	*Coefficient*	*GenDist*	*p-value*	*R2*	*p-value*
PB2	(Intercept)	6.15E-03	1	0.347	0.001
	GeogDist	1.77E-06	0.001		
	Year	2.82E-03	0.001		
PB1	(Intercept)	7.38E-03	1	0.351	0.001
	GeogDist	1.19E-06	0.001		
	Year	3.05E-03	0.001		
PA	(Intercept)	6.18E-03	1	0.336	0.001
	GeogDist	1.29E-06	0.001		
	Year	2.33E-03	0.001		
HA	(Intercept)	9.78E-03	1	0.419	0.001
	GeogDist	2.05E-06	0.001		
	Year	5.08E-03	0.001		
NP	(Intercept)	5.95E-03	1	0.216	0.001
	GeogDist	1.29E-06	0.001		
	Year	1.65E-03	0.001		
NA	(Intercept)	1.08E-02	1	0.136	0.001
	GeogDist	2.53E-06	0.001		
	Year	2.04E-03	0.001		
MP1	(Intercept)	5.98E-03	1	0.191	0.001
	GeogDist	1.60E-06	0.001		
	Year	2.06E-03	0.001		
NS1	(Intercept)	6.48E-03	1	0.365	0.001
	GeogDist	2.66E-06	0.001		
	Year	3.09E-03	0.001		

GenDist indicates the genetic distance variable, GeogDist indicates the geographic distance variable, Year indicates the temporal distance variable.

**Figure 4:**
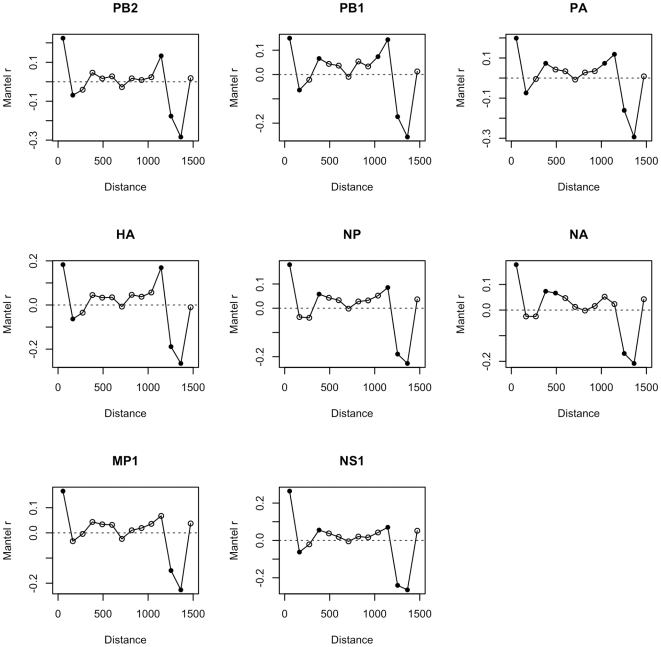
Mantel spatial correlograms, stratified by influenza gene segment. Correlograms show the relationship between geographic distance (x-axis) and the Mantel r correlation score (y-axis) of HK821-like HPAIVs. Under the null hypothesis of no relationship between geographic location and genetic similarity, all points would be on the zero line. Points above the zero line indicate lower genetic distance between case pairs. Points below the zero line indicate greater genetic distance between case pairs. Solid symbols are statistically significant, hollow symbols are not. The sharp rise to the furthest point in the correlograms is an artifact of edge effects caused by the spatial structure of the data, and does not indicate genetic similarity at the highest geographic distances between viruses.

Although the Mantel and MRM tests show that there is a significant relationship between genetic and geographic distance, the Mantel correlograms indicate that this relationship is not the same across all geographic distances (Figure 4). While the precise patterns of the correlograms vary by gene, the overall pattern is one of less genetic distance among viruses at scales of zero to approximately 1,100 km. At distances greater than 1,100 km, all genes exhibit strong and significant larger than expected genetic distances. Thus, compared to the null hypothesis of no relationship between geographic and genetic distance, measured genetic distances between case pairs are either somewhat less than expected at small spatial lags or significantly greater than expected at large spatial lags.

## Discussion

We characterized the geographic and temporal structure of molecular evolution of highly pathogenic H5N1 HK821-like avian influenza viruses in Vietnam. Our conclusions illustrate the relationships between space, time and genetics among Vietnamese H5N1 viruses in domestic poultry which extends beyond previous findings about the evolution of these H5N1 viruses in Vietnam [1]. A positive, statistically significant relationship between geographic, temporal and genetic distance exists for all eight influenza genes and across all four years of analysis.

H5N1 avian influenza incidence in Vietnam is bi-modal, as seen in the distribution of case pairs (Figure 1). The clustering of avian influenza in the north, surrounding Hanoi, and the south, surrounding Ho Chi Minh City, was observed in the dataset and has been indicated in other studies [21]. This bi-modality varies greatly by year and gene (Figure 2), however, revealing that some genes evolved and moved across the landscape at higher rates than others.

Previous studies have shown that H5N1 viruses appeared in northern Vietnam and spread to southern Vietnam, and that regional genetic mutation took place [10]. We chose to investigate these regional differences further by explicitly dividing the dataset into cases that took place within-region and those that spanned the distance between northern and southern Vietnam. The statistical (Table 1) and graphical results (Figure 3) indicate that year of incidence has a strong effect on the importance of region of incidence and gene in correlating with genetic change. In support of these findings, the results from the MRM analyses shows that the temporality on genetic evolution is important; they also indicate that, when controlling for the strong influence of time of incidence, the geographic distance between cases is a strong predictor of the genetic distance between cases.

Since very little is known about H5N1 in central Vietnam, and since our dataset consists primarily of isolates from the north and south, we cannot conclude whether the viruses were transmitted by passing over central Vietnam or rather moved gradually southward across the length of the country. The Mantel correlograms (Figure 4) demonstrate, however, that viral genetic mutation did not occur uniformly across geographic space. At distances of less than 1,100 km between viruses (approximately the distance between Hanoi and Ho Chi Minh City), the genetic sequences of viruses tended to be more similar, though the statistical significance of this similarity is affected by low numbers of observations at these middle distances. Case pairs that were greater than 1,100 km apart, however, exhibited high degrees of genetic dissimilarity. This dissimilarity was statistically significant for all eight genes. These results suggest that high levels of genetic dissimilarity are observed at large spatial scales (i.e. between north and south Vietnam), and that H5N1 influenza in Vietnam between 2003 and 2007 did not evolve gradually as it spread south, but rather that significant isolation by distance occurred. Viruses with similar ancestral lineages, e.g. the HK821-like viruses from China, evolve at different rates and in different ways in domestic poultry in northern compared to southern Vietnam, producing viruses that are genetically distant both within- and between-regions by 2007.

Our results clarify and improve understanding of how H5N1 HK821-like avian influenza evolves in space and time in poultry in Vietnam. We were able to observe the sequential establishment of closely related genotypes at two distinct population centers, and follow their differentiation due to relative isolation of the viral hubs over a period of several years. We found several patterns that suggest one general model of evolution in this viral system: 1) within regions, viral mixing in poultry moves toward heterogeneity and the emergence of local types; 2) differentiation was centered around regional viral hubs located at centers of human and bird population density; and 3) evolution occurs because of relative isolation of the hubs, most likely fed by the abundant supply of domesticated poultry (and people) at the hubs. The analysis thus suggests that at the scale of neighboring city hubs and the intervening hinterland, evolution of H5N1 follows the pattern described by classical theory of genetic differentiation due to isolation by distance [22], wherein gradual differential evolution of previously similar populations is driven by geographic distance and time. While the interactions between time, space and genetic evolution could potentially be circumvented by repeated introductions of H5N1 strains of different lineages into Vietnam, we would still expect to see the northern region around Hanoi and the southern region around Ho Chi Minh City acting as important sites of genetic differentiation rather than the central region of the country. Further investigation of the local-level ecosystems of northern versus southern Vietnam will shed light onto what human and environmental factors are driving the place-specific evolution of H5N1 avian influenza viruses.

## Materials and Methods

### Viral Genetic and Geographic Dataset

**Figure 5:**
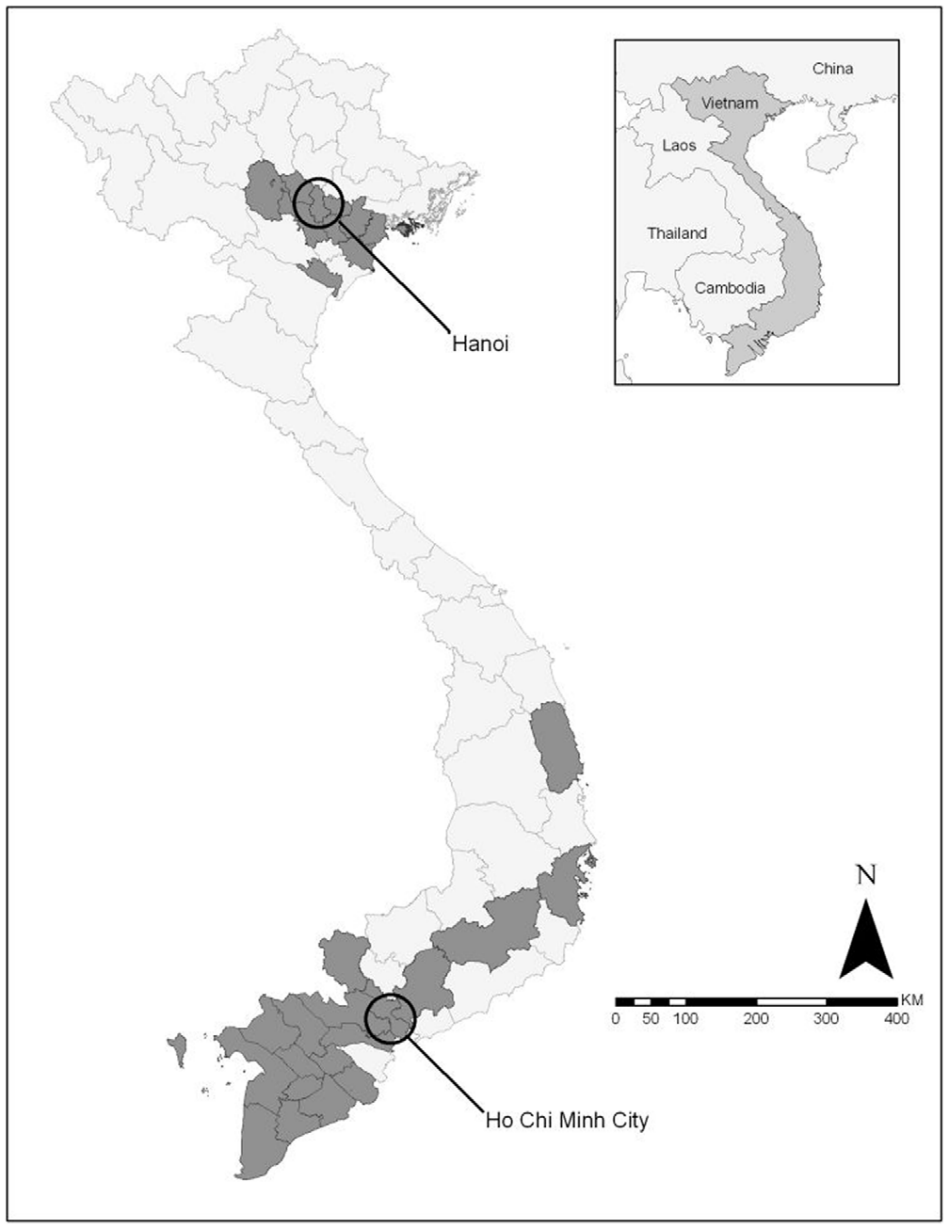
Geographic distribution of H5N1 highly pathogenic avian influenza viruses (HPAIVs) used in this study. Darkened provinces indicate locations of virus isolation.

The dataset consists of 125 H5N1 HPAIVs isolated from domestic poultry in Vietnam between 2003 and 2007, each of which has the full-length or nearly full-length genomic sequences for all 8 segments (Table S1) [10]. All eight genes in the viruses belong to the Clade1/VN3 (HK821-like) line. In the context of this work, an influenza gene is referred to a gene segment. These viruses have a wide geographic distribution and were isolated in 28 provinces primarily across northern and southern Vietnam, with a few isolates from Vietnam's central provinces (Figure 5). Each virus was assigned a unique identification number, allowing us to link geographic location, genetic sequence and temporal data in later analyses, and the dataset was sorted in ascending order by this unique ID. All matrices subsequently generated have identical ordering.

### Genetic, Geographic, and Temporal Distance Matrices Creation

In order to generate the genetic distance matrices, we constructed a maximum likelihood phylogenetic tree using nucleotide sequences as described before [10]. Then the patristic distance, which is the sum of branch length on a path between a pair of taxa in the phylogenetic tree, was extracted using software PATRISTIC [23]. Genetic distances were arranged into matrices for each of the eight influenza viral gene segments.

The database included the province for each virus isolate. The ID number and province listing for each of the 125 viruses was imported into ArcGIS geographic information systems (GIS) software and linked to a map of Vietnam's 64 provinces. Each virus was then assigned the latitude and longitude coordinates of the centroid of the province where the virus was isolated. While a more precise virus isolation location is preferable, only the province of incidence was available. While assigning viruses to the province centroid is somewhat arbitrary, the relatively small area of Vietnamese provinces makes this decision less problematic but does prevent within-province analysis. The list of viruses with their attached geographic location was then used to generate a geographic distance matrix. We calculated the ground distance in kilometers between viruses using the great circle distance measure, which takes the curvature of the earth's surface into account. Five geographic distance matrices were generated: 2003, 2004, 2005, 2007, and one for the entire study period.

Using the year of isolation and the unique ID numbers, a temporal distance matrix was created for all 125 viruses.

### Statistical Analysis

All matrices were analyzed with R2.7.1 [24]. Using the eight genetic and single geographic distance matrices, we generated distributional scatterplots and fit a least squares line. Potential differences in genetic versus geographic distance relationships according to gene segment led us to stratify and plot the data, first by gene then further by genes in individual years.

The clustering of cases geographically raised the question of whether differences in genetic distance between viruses exist for between-region case pairs versus within-region case pairs. To explore this possibility, we used a distance of 800 kilometers (approximately half the length of the country) to assign case pairs as either taking place within the same region (e.g., both in northern Vietnam) or between regions (i.e., one in the north, the other in the south). A three-way analysis of variance (ANOVA) measured genetic distance as a function of gene, year and within- versus between-region designation, testing whether underlying genetic distributions differ regionally. ANOVA can, in some cases, be a problematic test for non-independent matrix observations, but the inclusion of the region variable helps to overcome spatial dependence in the dataset. The statistical significance of the relationships we tested using the ANOVA, however, are rendered suspect by the dependence that exists between observations in a distance matrix. Boxplots complement the ANOVA results by showing the nature of the interaction between geographic lag (within- vs. between-region) and year in their explanation of genetic distance among viruses.

As is frequently the case with spatial data, there was dependence in the geographic distance matrix due to clustering of the sample locations. Additionally, matrix observations are non-independent, based upon paired datapoints. The findings from the ANOVA and boxplot results suggested that an interesting relationship between the molecular evolution of viruses and geographic space existed, so we implemented Mantel testing. Mantel tests are used to test for correlation between distance matrices when the underlying probability distribution of the test statistic is unknown and when dependence is present. Mantel tests overcome the lack of observation independence by randomly shuffling the values in one of the matrices multiple times and calculating correlations between the shuffled and original matrices. The probability distribution of the test statistic (the Mantel r) is generated by this random permutation process and used as a basis for assigning a probabilistic interpretation of the true correlation statistic between the observed response and predictor matrices. Mantel tests were conducted for each of the influenza virus' eight genes (aggregated by year), comparing genetic and geographic distance matrices.

We built multiple regression models using Mantel tests (MRM) to simultaneously but separately test the effect of time and space on genetic distance. MRM allows for the analysis of two or more matrices [25], where the response matrix (genetic distance) is regressed on multiple explanatory matrices (geographic and temporal distances), while also controlling for the effect of those explanatory matrices. In other words, MRM enabled us to determine the statistical significance and relative importance of each explanatory variable (space and time) individually while also acknowledging the effect of both.

Mantel tests indicate whether genetic and geographic distances are related, but they tell us little about the form of this relationship. In order to explore the question of how viral evolution occurs across space we generated Mantel correlograms (also known as spatial Mantel correlograms) stratified by gene. In a Mantel correlogram, the geographic distance between observations is divided into lags, and Mantel statistics (including significance) are calculated for case pairs who fall within each lag. Significance tests at each spatial lag are dependent on the sample size of points that fall within each threshold, so for distances where there were few observations (i.e. because of the bias in our dataset for sampling points in the north and south) the findings were statistically insignificant. Correlograms thus display the degree of likeness or difference among viruses at specific geographic distances. Points above the zero line exhibit positive autocorrelation, those below have negative autocorrelation. The Mantel correlograms allowed us to assess whether the degree of genetic dissimilarity among viruses corresponded to the scale of geographic distance between the viruses.

## Supporting Information

Table S1 Summary of the H5N1 AIVs used in this study.
(0.02 MB PDF)
