# Supplementary material for: Spatiotemporal Structure of Molecular Evolution of H5N1 Highly Pathogenic Avian Influenza Viruses in Vietnam
Source: PLoS One. 2010 Jan 8;5(1):e8631. doi: 10.1371/journal.pone.0008631 (PMC2799669; doi:10.1371/journal.pone.0008631)
Supplement: Table S1 — Summary of the H5N1 AIVs used in this study. (0.02 MB PDF) [file pone.0008631.s001.pdf]

Table S1. Summary of the H5N1 AIVs used in this study.

Year	Virus	Location	Accession Number							
			PB2	PB1	PA	HA	NP	NA	MP	NS
2003	Ck/VN/19/03	HA TAY	DQ492854	DQ493378	DQ493291	DQ497678	DQ493116	DQ493027	DQ492939	DQ493203
2003	Ck/VN/20/03	HA TAY	DQ492855	DQ493379	DQ493292	DQ497679	DQ493117	DQ493028	DQ492940	DQ493204
2003	Ck/VN/27/03	HA TAY	DQ320872	DQ321332	DQ321266	DQ320938	DQ321134	DQ321069	DQ321003	DQ321200
2003	Ck/VN/28/03	HA TAY	DQ492857	DQ493381	DQ493294	DQ497681	DQ493119	DQ493030	DQ492942	DQ493206
2003	Ck/VN/30/03	HA TAY	DQ492858	DQ493382	DQ493295	DQ497682	DQ493120	DQ493031	DQ492943	DQ493207
2003	Ck/VN/4/03	VINH PHUC	DQ492867	DQ493391	DQ493304	DQ497691	DQ493129	DQ493040	DQ492952	DQ493216
2003	Ck/VN/5/03	VINH PHUC	DQ492868	DQ493392	DQ493305	DQ497692	DQ493130	DQ493041	DQ492953	DQ493217
2003	Ck/VN/8/03	VINH PHUC	DQ492869	DQ493393	DQ493306	DQ497693	DQ493131	DQ493042	DQ492954	DQ493218
2003	Dk/VN/15/03	HA NOI	DQ492846	DQ493370	DQ493285	DQ497670	DQ493108	DQ493019	DQ492931	DQ493195
2003	Dk/VN/17/03	HA NOI	DQ492848	DQ493372	DQ493286	DQ497672	DQ493110	DQ493021	DQ492933	DQ493197
2003	mallard/VN/16/03	HA NOI	DQ492847	DQ493371	DQ493284	DQ497671	DQ493109	DQ493020	DQ492932	DQ493196
2003	mallard/VN/21/03	HA TAY	DQ492856	DQ493380	DQ493293	DQ497680	DQ493118	DQ493029	DQ492941	DQ493205
2003	mallard/VN/3/03	VINH PHUC	DQ492866	DQ493390	DQ493303	DQ497690	DQ493128	DQ493039	DQ492951	DQ493215
2003	Ck/VN/NCVD7/03	VINH PHUC	ISDN40366	ISDN40848	ISDN40949	ISDN38690	ISDN40075	ISDN38698	ISDN39966	ISDN40023
2003	Dk/VN/NCVD29/03	HA TAY	ISDN40375	ISDN40856	ISDN40946	ISDN40340	ISDN45791	ISDN38711	ISDN45807	ISDN45784
2003	M.Dk/VN/NCVD14/03	VINH PHUC	ISDN40369	ISDN40851	ISDN40944	ISDN40332	ISDN40080	ISDN38699	ISDN39972	ISDN40029
2003	M.Dk/VN/NCVD21/03	HUNG YEN	ISDN40371	ISDN40853	ISDN40942	ISDN38691	ISDN40082	ISDN38700	ISDN39979	ISDN40036
2003	M.Dk/VN/NCVD28/03	HA TAY	ISDN40374	ISDN40855	ISDN40941	ISDN40913	ISDN40084	ISDN38701	ISDN45806	ISDN40038
2004	Ck/VN/132/04	VINH LONG	DQ492892	DQ493417	DQ493331	DQ497718	DQ493155	DQ493067	DQ492979	DQ493243
2004	Ck/VN/133/04	CAN THO	DQ492875	DQ493399	DQ493313	DQ497700	DQ493138	DQ493049	DQ492961	DQ493225
2004	Ck/VN/135/04	BAC LIEU	DQ492871	DQ493395	DQ493308	DQ497695	DQ493133	DQ493044	DQ492956	DQ493220
2004	Ck/VN/147/04	CAN THO	DQ492876	DQ493400	DQ493314	DQ497701	DQ493139	DQ493050	DQ492962	DQ493226
2004	Ck/VN/149/04	BAC LIEU	DQ492872	DQ493396	DQ493309	DQ497696	DQ493134	DQ493045	DQ492957	DQ493221
2004	Ck/VN/159/04	SOC TRANG	DQ492888	DQ493412	DQ493326	DQ497713	DQ493150	DQ493062	DQ492974	DQ493238
2004	Ck/VN/260/04	HAU GIANG	DQ492881	DQ493405	DQ493319	DQ497706	DQ493144	DQ493055	DQ492967	DQ493231
2004	Ck/VN/32/04	THAI BINH	DQ492863	DQ493387	DQ493300	DQ497687	DQ493125	DQ493036	DQ492948	DQ493212
2004	Ck/VN/52/04	BINH DINH	DQ492874	DQ493398	DQ493311	DQ497698	DQ493136	DQ493047	DQ492959	DQ493223
2004	Ck/VN/53/04	KHANH HOA	DQ492885	DQ493409	DQ493323	DQ497710	DQ493147	DQ493059	DQ492971	DQ493235
2004	Ck/VN/AG-010/04	AN GIANG	DQ138170	DQ138149	AY724790	AY724789	DQ099765	DQ094278	DQ094256	AY770612
2004	Ck/VN/CT-018/04	CAN THO	DQ138172	DQ138151	DQ099786	AY724793	DQ099767	DQ094280	DQ094258	AY770614
2004	Ck/VN/DN-045/04	DONG NAI	DQ138176	DQ138155	AY724786	AY724785	DQ099771	DQ094284	DQ094262	AY770618
2004	Ck/VN/DT-015/04	DONG THAP	DQ138171	DQ138150	AY724792	AY724791	DQ099766	DQ094279	DQ094257	AY770613
2004	Ck/VN/DT171/04	DONG THAP	DQ320871	DQ321331	DQ321265	DQ320937	DQ321133	DQ321068	DQ321006	DQ321199
2004	Ck/VN/DT-171/04	DONG THAP	DQ138177	DQ138156	DQ099788	DQ099759	DQ099772	AY720943	DQ094263	AY770619
2004	Ck/VN/HCM-022/04	HOU CHI MING	DQ138175	DQ138154	AY724784	AY724783	DQ099770	DQ094283	DQ094261	AY770617
2004	Ck/VN/LA-024/04	LONG AN	DQ138174	DQ138153	AY724796	AY724795	DQ099769	DQ094282	DQ094260	AY770616
2004	Ck/VN/LD-080/04	LAM DONG	DQ138178	DQ138158	DQ099790	DQ099760	DQ099774	DQ094287	DQ094265	AY770621
2004	Ck/VN/TG-023/04	TIEN GIANG	DQ138173	DQ138152	DQ099787	DQ099758	DQ099768	DQ094281	DQ094259	AY770615
2004	Ck/VN/TN-025/04	TAY NINH	DQ138166	DQ138145	DQ099784	DQ099755	DQ099761	DQ094276	DQ094252	AY770609
2004	Ck/VN/VL-008/04	VINH LONG	DQ138169	DQ138148	AY724788	AY724787	DQ099764	DQ094277	DQ094255	AY770611
2004	Dk/VN/148/04	CAN THO	DQ492877	DQ493401	DQ493315	DQ497702	DQ493140	DQ493051	DQ492963	DQ493227
2004	Dk/VN/219/04	HA TAY	DQ492860	DQ493384	DQ493297	DQ497684	DQ493122	DQ493033	DQ492945	DQ493209
2004	Dk/VN/220/04	HA TAY	DQ492861	DQ493385	DQ493298	DQ497685	DQ493123	DQ493034	DQ492946	DQ493210
2004	Dk/VN/258/04	TRA VINH	DQ492893	DQ493415	DQ493329	DQ497716	DQ493153	DQ493065	DQ492977	DQ493241
2004	Dk/VN/40/04	HA NOI	DQ492849	DQ493373	DQ493287	DQ497673	DQ493111	DQ493022	DQ492934	DQ493198
2004	Dk/VN/48/04	HA TAY	DQ492859	DQ493383	DQ493296	DQ497683	DQ493121	DQ493032	DQ492944	DQ493208
2004	Dk/VN/N-XX/04	HA TAY	DQ492862	DQ493386	DQ493299	DQ497686	DQ493124	DQ493035	DQ492947	DQ493211
2004	Dk/VN/TG-007A/04	TIEN GIANG	DQ138167	DQ138146	AY720947	DQ099756	DQ099762	AY720946	DQ094253	AY770610
2004	Gs/VN/264/04	HAU GIANG	DQ492882	DQ493406	DQ493320	DQ497707	DQ493145	DQ493056	DQ492968	DQ493232
2004	quail/VN/177/04	TRA VINH	DQ492890	DQ493414	DQ493328	DQ497715	DQ493152	DQ493064	DQ492976	DQ493240

2004	VN/CL01/04	HOU CHI MING	DQ492894	DQ493418	DQ493332	DQ497719	DQ493156	DQ250159	DQ492980	DQ493244
2004	VN/CL100/04	TAY NINH	DQ492898	DQ493424	DQ493337	DQ497725	DQ493162	DQ250162	DQ492986	DQ493250
2004	VN/CL26/04	LAM DONG	DQ492896	DQ493422	DQ493335	DQ497723	DQ493160	DQ493072	DQ492984	DQ493248
2004	VN/CL36/04	BINH DINH	DQ492897	DQ493423	DQ493336	DQ497724	DQ493161	DQ250161	DQ492985	DQ493249
2004	Ck/VN/NCVD31/04	HA TAY	ISDN40377	ISDN40858	ISDN40950	EF541406	ISDN41056	ISDN38702	ISDN45754	ISDN45785
2005	Ck/VN/348/05	HA NOI	DQ492852	DQ493376	DQ493289	DQ497676	DQ493114	DQ493025	DQ492937	DQ493201
2005	Ck/VN/393/05	KIEN GIANG	DQ492887	DQ493411	DQ493325	DQ497712	DQ493149	DQ493061	DQ492973	DQ493237
2005	Ck/VN/398/05	DONG THAP	DQ492878	DQ493402	DQ493316	DQ497703	DQ493141	DQ493052	DQ492964	DQ493228
2005	Dk/VN/272/05	HA NOI	DQ492850	DQ493374	DQ493288	DQ497674	DQ493112	DQ493023	DQ492935	DQ493199
2005	Dk/VN/283/05	HAU GIANG	DQ492883	DQ493407	DQ493321	DQ497708	DQ493107	DQ493057	DQ492969	DQ493233
2005	Dk/VN/286/05	BAC LIEU	DQ492873	DQ493397	DQ493310	DQ497697	DQ493135	DQ493046	DQ492958	DQ493222
2005	Dk/VN/317/05	THAI BINH	DQ492865	DQ493389	DQ493302	DQ497689	DQ493127	DQ493038	DQ492950	DQ493214
2005	Dk/VN/376/05	TRA VINH	DQ492879	DQ493403	DQ493317	DQ497704	DQ493142	DQ493053	DQ492965	DQ493229
2005	Dk/VN/543/05	HAU GIANG	DQ492891	DQ493416	DQ493330	DQ497717	DQ493154	DQ493066	DQ492978	DQ493242
2005	Dk/VN/N-TB/05	THAI BINH	DQ492864	DQ493388	DQ493301	DQ497688	DQ493126	DQ493037	DQ492949	DQ493213
2005	Dk/VN/S640/05	AN GIANG	DQ492870	DQ493394	DQ493307	DQ497694	DQ493132	DQ493043	DQ492955	DQ493219
2005	Dk/VN/S654/05	CAN THO	DQ320863	DQ321335	DQ321269	DQ320936	DQ321137	DQ321067	DQ321002	DQ321203
2005	mallard/VN/347/05	HA NOI	DQ492851	DQ493375	DQ493283	DQ497675	DQ493113	DQ493024	DQ492936	DQ493200
2005	mallard/VN/352/05	HA NOI	DQ492853	DQ493377	DQ493290	DQ497677	DQ493115	DQ493026	DQ492938	DQ493202
2005	quail/VN/282/05	KIEN GIANG	DQ492886	DQ493410	DQ493324	DQ497711	DQ493148	DQ493060	DQ492972	DQ493236
2005	VN/CL105/05	TRA VINH	DQ492899	DQ493425	DQ493338	DQ497726	DQ493163	DQ493075	DQ492987	DQ493251
2005	VN/CL115/05	VINH LONG	DQ492900	DQ493426	DQ493339	DQ497727	DQ493164	DQ250163	DQ492988	DQ493252
2005	VN/CL119/05	DONG THAP	DQ492901	DQ493427	DQ493340	DQ497728	DQ493165	DQ250164	DQ492989	DQ493253
2005	VN/CL2009/05	DONG THAP	DQ492902	DQ493428	DQ493341	DQ497729	DQ493166	DQ250165	DQ492990	DQ493254
2005	wildbird/VN/434/05	DONG THAP	DQ492880	DQ493404	DQ493318	DQ497705	DQ493143	DQ493054	DQ492966	DQ493230
2005	Ck/VN/NCVDCDC1/05	HA TAY	ISDN131304	ISDN131249	ISDN125522	ISDN124063	ISDN125681	ISDN124098	ISDN125576	ISDN125624
2005	Ck/VN/NCVDCDC18/05	HA NOI	ISDN131316	ISDN131264	ISDN125537	ISDN124076	ISDN125697	ISDN124111	ISDN125589	ISDN125641
2005	Ck/VN/NCVDCDC19/05	HA NOI	ISDN131317	ISDN131265	ISDN125538	ISDN124077	ISDN125698	ISDN124112	ISDN125590	ISDN125642
2005	Ck/VN/NCVDCDC22/05	HA NOI	ISDN131319	ISDN131267	ISDN125539	ISDN124078	ISDN125699	ISDN124113	ISDN125591	ISDN125645
2005	Ck/VN/NCVDCDC25/05	HAI DUONG	ISDN131321	ISDN131269	ISDN125541	ISDN124155	ISDN125701	ISDN124167	ISDN125593	ISDN125647
2005	Ck/VN/NCVDCDC27/05	BAC NINH	ISDN131323	ISDN131271	ISDN125543	ISDN124080	ISDN125703	ISDN124115	ISDN125594	ISDN125649
2005	Ck/VN/NCVDCDC3/05	HA TAY	ISDN131306	ISDN131251	ISDN125524	ISDN124065	ISDN125683	ISDN124100	ISDN125578	ISDN125626
2005	Ck/VN/NCVDCDC37/05	PHU THO	ISDN131329	ISDN131281	ISDN125550	ISDN124085	ISDN125713	ISDN124120	ISDN125602	ISDN125658
2005	Ck/VN/NCVDCDC38/05	THAI BINH	ISDN131330	ISDN131282	ISDN125551	ISDN124086	ISDN125714	ISDN124121	ISDN125603	ISDN125659
2005	Ck/VN/NCVDCDC40/05	HAI PHONG	ISDN131332	ISDN131284	ISDN125553	ISDN124088	ISDN125716	ISDN124123	ISDN125605	ISDN125661
2005	Ck/VN/NCVDCDC42/05	HAI PHONG	ISDN131334	ISDN131286	ISDN125555	ISDN124090	ISDN125718	ISDN124125	ISDN125607	ISDN125663
2005	Ck/VN/NCVDCDC43/05	HAI PHONG	ISDN131335	ISDN131287	ISDN125556	ISDN124091	ISDN125719	ISDN124126	ISDN125608	ISDN125664
2005	Ck/VN/NCVDCDC52/05	VINH PHUC	ISDN131337	ISDN131296	ISDN125563	ISDN124160	ISDN125728	ISDN124172	ISDN125617	ISDN125673
2005	Dk/VN/NCVDCDC10/05	AN GIANG	ISDN131310	ISDN131256	ISDN125529	ISDN124069	ISDN125689	ISDN124104	ISDN125582	ISDN125633
2005	Dk/VN/NCVDCDC11/05	AN GIANG	ISDN131311	ISDN131257	ISDN125530	ISDN124070	ISDN125690	ISDN124105	ISDN125583	ISDN125634
2005	Dk/VN/NCVDCDC12/05	AN GIANG	ISDN131312	ISDN131258	ISDN125531	ISDN124071	ISDN125691	ISDN124106	ISDN125584	ISDN125635
2005	Dk/VN/NCVDCDC14/05	TIEN GIANG	ISDN131314	ISDN131260	ISDN125533	ISDN124073	ISDN125693	ISDN124108	ISDN125586	ISDN125637
2005	Dk/VN/NCVDCDC2/05	HA TAY	ISDN131305	ISDN131250	ISDN125523	ISDN124064	ISDN125682	ISDN124099	ISDN125577	ISDN125625
2005	Dk/VN/NCVDCDC39/05	THAI BINH	ISDN131331	ISDN131283	ISDN125552	ISDN124087	ISDN125715	ISDN124122	ISDN125604	ISDN125660
2005	Dk/VN/NCVDCDC41/05	HAI PHONG	ISDN131333	ISDN131285	ISDN125554	ISDN124089	ISDN125717	ISDN124124	ISDN125606	ISDN125662
2005	Dk/VN/NCVDCDC63/05	AN GIANG	ISDN131346	ISDN127528	ISDN127541	ISDN127554	ISDN127566	ISDN127579	ISDN127592	ISDN127605
2005	Dk/VN/NCVDCDC64/05	NINH BINH	ISDN127517	ISDN127529	ISDN127542	ISDN127555	ISDN127567	ISDN127580	ISDN127593	ISDN127606
2005	ENV/VN/NCVDCDC54/05	HA TAY	ISDN131339	ISDN131298	ISDN125564	ISDN124161	ISDN125730	ISDN124173	ISDN125619	ISDN125675
2005	ENV/VN/NCVDCDC55/05	VINH PHUC	ISDN131340	ISDN131299	ISDN125565	ISDN124097	ISDN125731	ISDN124132	ISDN125680	ISDN125676
2005	ENV/VN/NCVDCDC56/05	TIEN GIANG	ISDN131341	ISDN131300	ISDN125566	ISDN124162	ISDN125732	ISDN124174	ISDN125620	ISDN125677
2007	Ck/VN/NCVD-10/07	BAC LIEU	CY030308	CY030309	CY030310	CY030311	CY030312	CY030313	CY030314	CY030315
2007	Ck/VN/NCVD-15/07	HAU GIANG	CY030340	CY030341	CY030342	CY030343	CY030344	CY030345	CY030346	CY030347
2007	Ck/VN/NCVD-21/07	HA TAY	CY030388	CY030389	CY030390	CY030391	CY030392	CY030393	CY030394	CY030395
2007	Ck/VN/NCVD-24/07	HAU GIANG	CY030412	CY030413	CY030414	CY030415	CY030416	CY030417	CY030418	CY030419

2007	Ck/VN/NCVD-3/07	CA MAU	CY030252	CY030253	CY030254	CY030255	CY030256	CY030257	CY030258	CY030259
2007	Dk/VN/NCVD-1/07	CA MAU	CY030236	CY030237	CY030238	CY030239	CY030240	CY030241	CY030242	CY030243
2007	Dk/VN/NCVD-12/07	HAU GIANG	CY030324	CY030325	CY030326	CY030327	CY030328	CY030329	CY030330	CY030331
2007	Dk/VN/NCVD-13/07	HAU GIANG	CY030332	CY030333	CY030334	CY030335	CY030336	CY030337	CY030338	CY030339
2007	Dk/VN/NCVD-16/07	KIEN GIANG	CY030348	CY030349	CY030350	CY030351	CY030352	CY030353	CY030354	CY030355
2007	Dk/VN/NCVD-17/07	KIEN GIANG	CY030356	CY030357	CY030358	CY030359	CY030360	CY030361	CY030362	CY030363
2007	Dk/VN/NCVD-18/07	KIEN GIANG	CY030364	CY030365	CY030366	CY030367	CY030368	CY030369	CY030370	CY030371
2007	Dk/VN/NCVD-19/07	KIEN GIANG	CY030372	CY030373	CY030374	CY030375	CY030376	CY030377	CY030378	CY030379
2007	Dk/VN/NCVD-2/07	CA MAU	CY030244	CY030245	CY030246	CY030247	CY030248	CY030249	CY030250	CY030251
2007	Dk/VN/NCVD-25/07	SOC TRANG	CY030420	CY030421	CY030422	CY030423	CY030424	CY030425	CY030426	CY030427
2007	Dk/VN/NCVD-26/07	VINH LONG	CY030428	CY030429	CY030430	CY030431	CY030432	CY030433	CY030434	CY030435
2007	Dk/VN/NCVD-30/07	KIEN GIANG	CY030443	CY030444	CY030722	CY030445	CY030446	CY030447	CY030448	CY030449
2007	Dk/VN/NCVD-6/07	BAC LIEU	CY030276	CY030277	CY030278	CY030279	CY030280	CY030281	CY030282	CY030283
2007	Dk/VN/NCVD-7/07	BAC LIEU	CY030284	CY030285	CY030286	CY030287	CY030288	CY030289	CY030290	CY030291
2007	Dk/VN/NCVD-8/07	BAC LIEU	CY030292	CY030293	CY030294	CY030295	CY030296	CY030297	CY030298	CY030299
2007	Dk/VN/NCVD-9/07	BAC LIEU	CY030300	CY030301	CY030302	CY030303	CY030304	CY030305	CY030306	CY030307
2007	M.Dk/VN/NCVD-11/07	BAC LIEU	CY030316	CY030317	CY030318	CY030319	CY030320	CY030321	CY030322	CY030323
2007	M.Dk/VN/NCVD-23/07	CA MAU	CY030404	CY030405	CY030406	CY030407	CY030408	CY030409	CY030410	CY030411
2007	M.Dk/VN/NCVD-29/07	CA MAU	CY030436	CY030437	CY030721	CY030438	CY030439	CY030440	CY030441	CY030442
2007	M.Dk/VN/NCVD-4/07	CA MAU	CY030260	CY030261	CY030262	CY030263	CY030264	CY030265	CY030266	CY030267
2007	M.Dk/VN/NCVD-5/07	CA MAU	CY030268	CY030269	CY030270	CY030271	CY030272	CY030273	CY030274	CY030275
